# Supplementary material for: De novo profile generation based on sequence context specificity with the long short-term memory network
Source: BMC Bioinformatics. 2018 Jul 18;19:272. doi: 10.1186/s12859-018-2284-1 (PMC6052547; doi:10.1186/s12859-018-2284-1)
Supplement: Supplementary file 1 — Figure S1. Learning curves of the LSTM, Figure S2. ROC curves of similarity search for the target (HHBlits) and predictors, Figure S3. Comparison of profile generation time with simulation data, Figure S4. ROC curves of the similarity search for each iterative method, Table S1. Comparison of pAUC values for SCOP classes for SCOP20 test datasets. (PDF 857 kb) [file 12859_2018_2284_MOESM1_ESM.pdf]

Sequence analysis

Supplemental data for "De novo profile generation based on sequence context specificity with the long short-term memory network"

Kazunori D Yamada[1,2] and Kengo Kinoshita[1,3,4*]

[1]Graduate School of Information Sciences, Tohoku University, Sendai, Japan, [2]Artificial Intelligence Research Center, National Institute of Advanced Industrial Science and Technology (AIST), Tokyo, Japan, [3]Tohoku Medical Megabank Organization, Tohoku University, Sendai, Japan, [4]Institute of Development, Aging, and Cancer, Tohoku University, Sendai, Japan

Table of contents

Figure S1: Learning curves of the LSTM.

Figure S2: ROC curves of similarity search for the target (HHBlits) and predictors.

Figure S3: Comparison of profile generation time with simulation data.

Figure S4: ROC curves of the similarity search for each iterative method.

Table S1: Comparison of pAUC values for SCOP classes for SCOP20 test datasets.

References

Figure S1

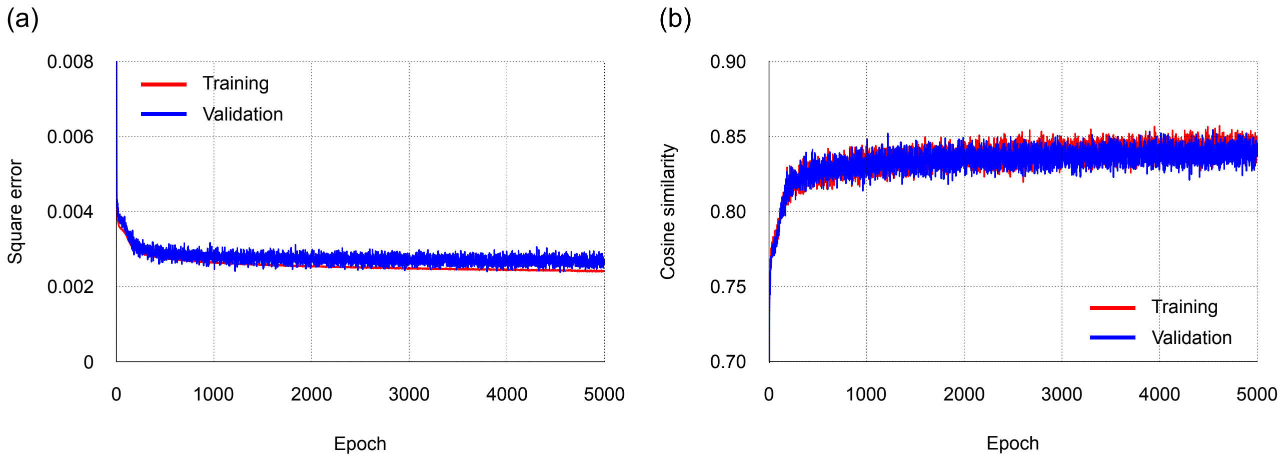

Learning curves of the LSTM. The red and blue lines represent training and validation curves, respectively. (a) Square errors between target and output vectors are shown. (b) Cosine similarity (accuracy) between target and output vectors is shown. Since there was no dissociation between training and validation curves, overfitting did not occur by 5,000 epochs.

Figure S2

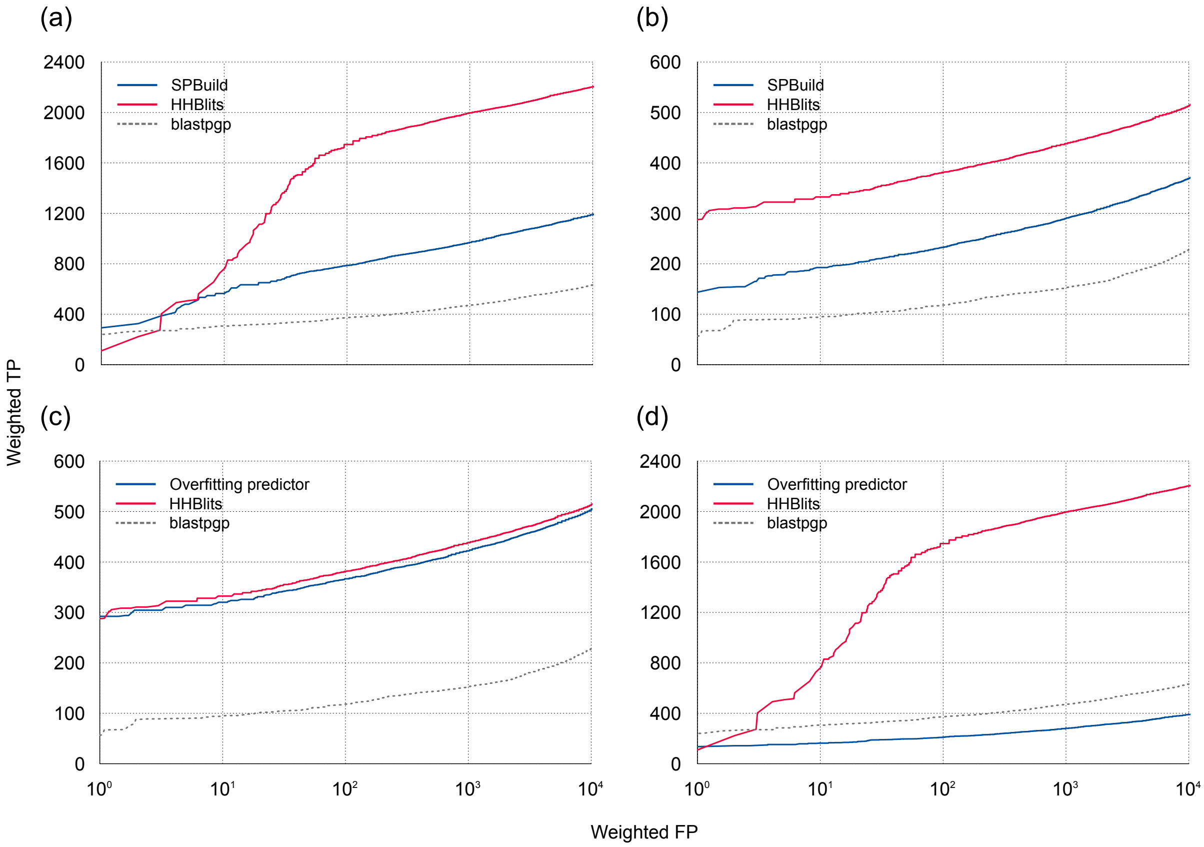

ROC curves of similarity search for the target (HHBlits) and predictors. The performance of blastpgp is shown for reference. Note that HHBlits does not indicate the original HHBlits searches because the profiles generated by HHBlits were used as input for PSI-BLAST in this study (see section 2.3 for details). (a) Sensitivity of HHBlits and SPBuild in the SCOP20 test dataset (5,819 sequences). SPBuild was trained on the integrated dataset of Pfam40 (1,601,009 sequences) and the SCOP20 learning dataset (1,329 sequences). The performance of SPBuild did not reach that of HHBlits. (b) Sensitivity of HHBlits and SPBuild in the SCOP20 learning dataset. The performance of SPBuild did not reach that of HHBlits. (c) Sensitivity of HHBlits and overfitting predictor in the SCOP20 learning dataset. The overfitting predictor was trained on the SCOP20 learning dataset. The performance of the predictor was almost the same as that of HHBlits (possibly overfitted). (d) Sensitivity of HHBlits and the overfitting predictor in the SCOP20 test dataset. The performance of the overfitting predictor did not reach that of blastpgp (sequence-sequence alignment method) because the predictor was overfitted to the SCOP20 learning dataset.

Figure S3

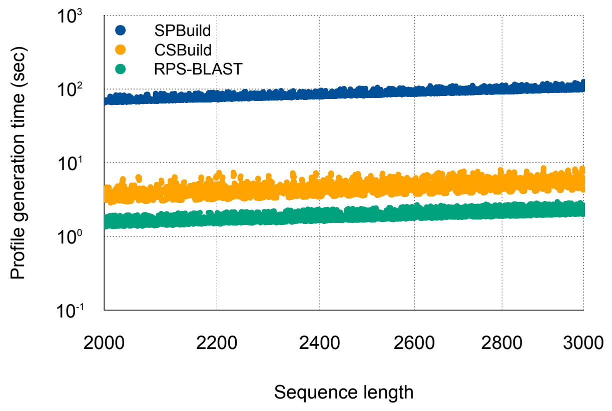

Comparison of profile generation time with simulation data. In total, 10,000 simulation sequence data were generated based on the background probability of the SCOP20 test dataset. The sequence length ranged from 2,001 to 3,000, and for each length, 10 sequences were generated. The profile generation time of each method was calculated using the dataset.

Figure S4

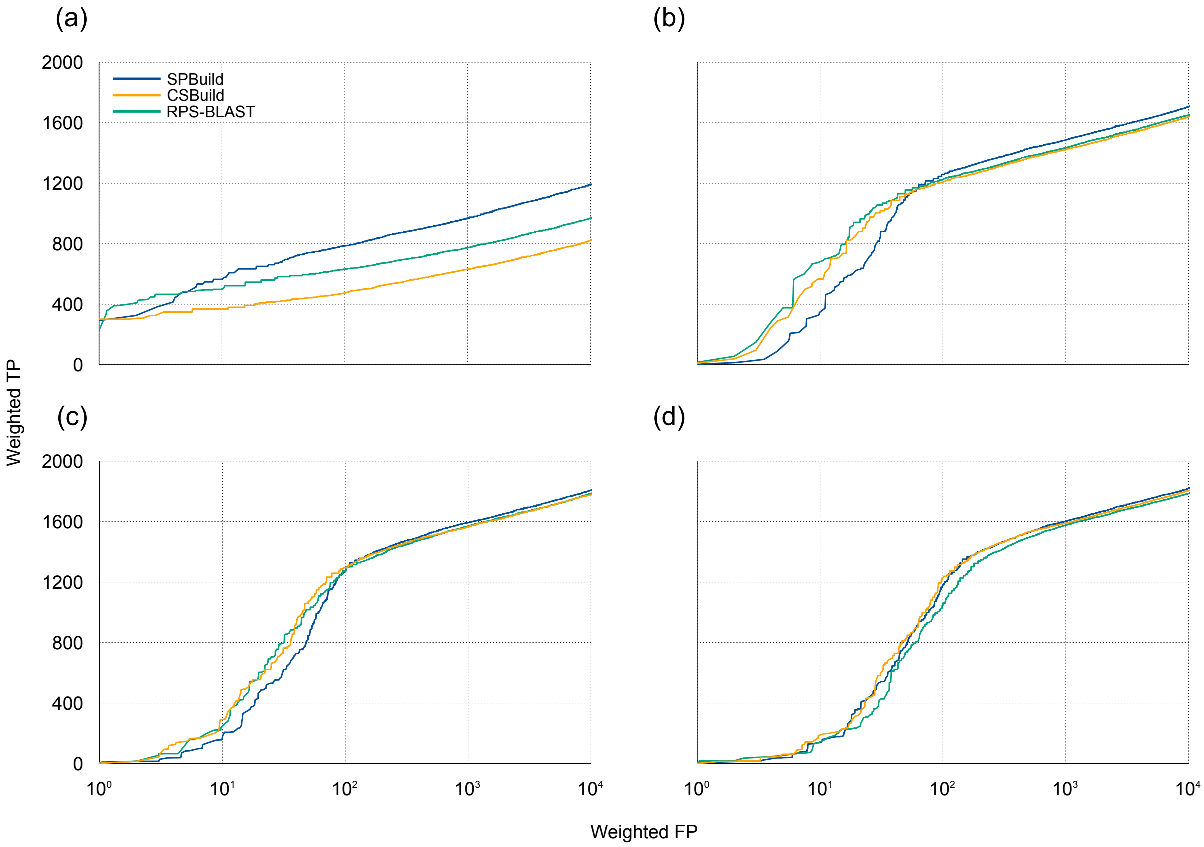

ROC curves of the similarity search for each iterative method. Profile files for all 5,819 sequences in the SCOP20 test dataset [1] were generated by each method and subsequently PSI-BLAST [2] searches with the profiles were conducted by psiblast version 2.2.30+ (SPBuild and RPS-BLAST) and blastpgp version 2.2.26 (CSBuild). As parameters of PSI-BLAST search, the iteration number, iteration database, inclusion e-values threshold, and inclusion sequence number were set to [1, 3, 5, or 7], UniRef30, 0.1, and 1,000, respectively. As an iteration database, UniRef30 was utilized. We evaluated one (a), three (b), five (c), and seven iterations (d). The pAUC values of SPBuild, CSBuild, and RPS-BLAST for three iterations were 0.322, 0.310, and 0.313, respectively; those for five iterations were 0.339, 0.337, and 0.336, respectively; and those for seven iterations were 0.336, 0.336, and 0.326, respectively.

Table S1

	SPBuild	CSBuild	RPS-BLAST	(HHBlits)
All	0.217	0.140	0.174	(0.451)
a	0.0390	0.0280	0.0333	(0.0773)
b	0.0705	0.0352	0.0473	(0.133)
c	0.0733	0.0520	0.0560	(0.143)
d	0.0536	0.0389	0.0523	(0.110)
Others	0.0156	0.0136	0.0144	(0.0270)

Comparison of pAUC values for SCOP classes for SCOP20 test datasets. The each SCOP class (a, b, c, d, and Others [e, f, and g]) consisted of 1,248, 1,416, 1,263, 1,427, and 465 (94, 110, and 261) sequences, respectively.

References

[1] Christof Angermüller, Andreas Biegert, and Johannes Söding. Discriminative modelling of context-specific amino acid substitution probabilities. *Bioinformatics*, 28:3240–3247, December 2012.

[2] Stephen F Altschul, Thomas L Madden, Alejandro A Schäffer, Jinghui Zhang, Zheng Zhang, Webb Miller, and David J Lipman. Gapped blast and psi-blast: a new generation of protein database search programs. *Nucleic acids research*, 25:3389–3402, September 1997.
